# The utility of the aortogram: contrast staining from an undiagnosed chronic aortic dissection

**DOI:** 10.1093/ehjcr/ytaf144

**Published:** 2025-03-26

**Authors:** Flavia Tejada Frisancho, Ryan Wallace, Giorgio Medranda, Brian Case

**Affiliations:** Department of Interventional Cardiology, MedStar Washington Hospital Center, 110 Irving St NW, 20010 Washington, DC, USA; Department of Interventional Cardiology, MedStar Washington Hospital Center, 110 Irving St NW, 20010 Washington, DC, USA; Department of Interventional Cardiology, NYU Langone Hospital – Long Island, 259 1st St, Mineola 11501, NY, USA; Department of Interventional Cardiology, MedStar Washington Hospital Center, 110 Irving St NW, 20010 Washington, DC, USA

A 74-year-old female with a past medical history of well-controlled hypertension, hyperlipidaemia, and end-stage renal disease on haemodialysis presenting for elective cardiac catheterization as part of her pre-renal transplant workup. The only symptom the patient endorsed was progressive dyspnoea on exertion; otherwise, she denied any chest pain. She denied any family history of aortic disease. During coronary angiography, contrast retention was noted in the ascending aorta, prompting an aortogram that demonstrated a large, saccular outpouching of the greater curvature suspected to be an aneurysm or healed aortic dissection (*Figure 1A–C*; Supplementary material online, *Videos S1* and *S2*). The patient underwent urgent computed tomography that confirmed a chronic Type A dissection extending along the ascending aorta from the sinotubular junction just over top of the right sinus of Valsalva terminating proximal to the common takeoff of the left brachiocephalic and common left carotid branches (*Figure 1D–F*). The patient underwent urgent surgery with subsequent intraoperative transoesophageal echocardiogram delineating the true and false lumen in the ascending aorta (*Figure 1G*; Supplementary material online, *Video S3*). The patient then underwent successful surgical ascending aorta replacement, hemiarch replacement, and reconstruction of aortic root with pericardium (*Figure 1H* and *I*). The postoperative course was complicated by atrial fibrillation, gastrointestinal bleeding, and general deconditioning. She was ultimately discharged to an acute rehabilitation centre.

**Figure 1:**
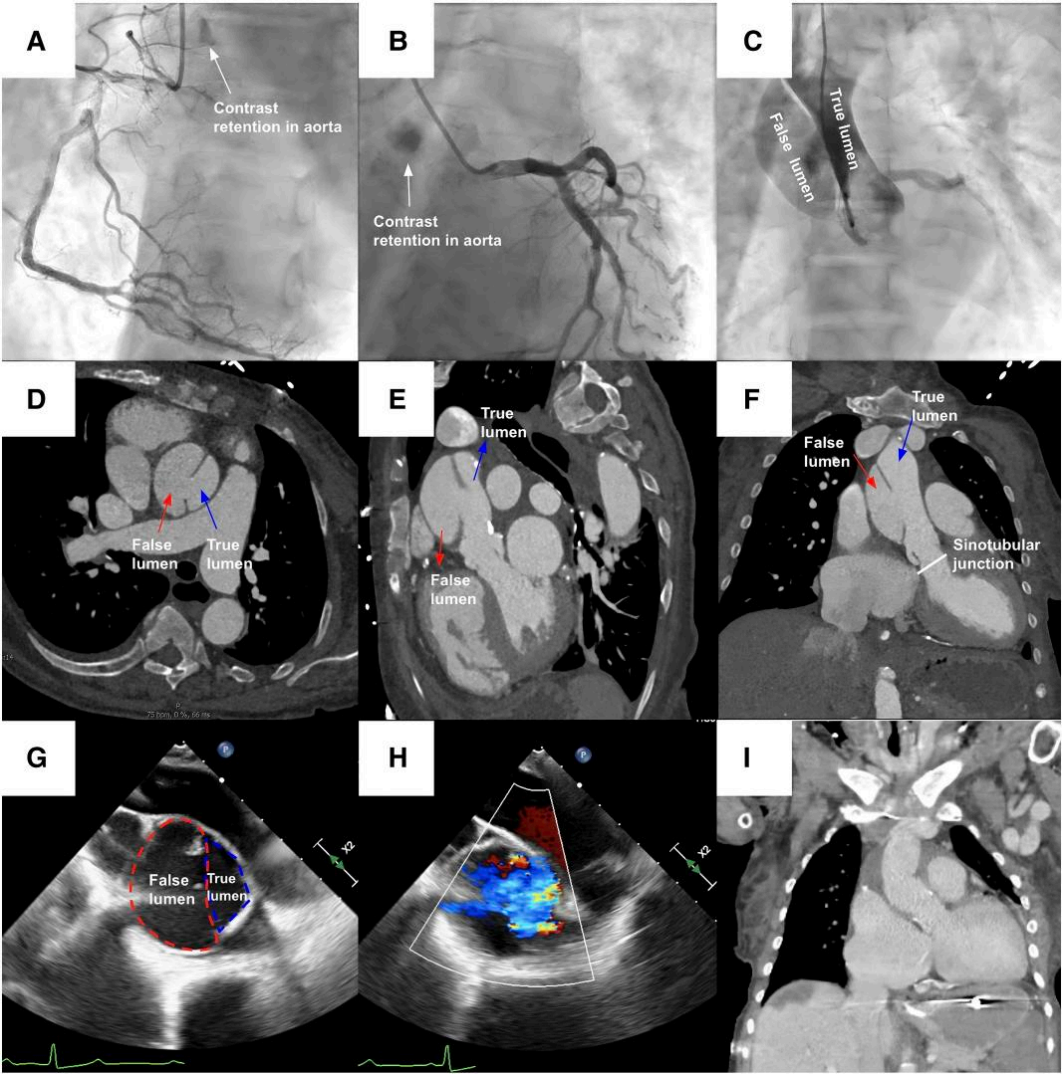
Chronic aortic dissection. Coronary angiogram images show the right coronary artery with contrast hang-up on aorta (*A*, *B*; arrows). Aortogram demonstrating pigtail catheter traversing the true lumen adjacent to the false lumen (*C*). Computed tomography angiography demonstrating aortic dissection originating just distal to the sinotubular junction and involving most of the proximal ascending aorta in axial, sagittal, and coronal views showing the true and false lumens (arrows) (*D–F*). Intraoperative transoesophageal echocardiogram showing proximal aorta and colour Doppler identifying the true lumen (*G*, *H*). Postoperative computed tomography angiography demonstrating successful ascending aorta replacement, hemiarch replacement, and reconstruction of aortic root with pericardium.

Type A aortic dissections are regarded as surgical emergencies because of the high risk of life-threatening complications, including cardiac tamponade, severe aortic regurgitation, myocardial infarction, or rupture of the aorta. Patients with untreated or late presenting Type A dissection rarely survive the acute event, making chronic dissections of the ascending aorta extremely uncommon.^1^ Chronic Type A aortic dissections that remain unrepaired adverse aortic events such as aortic rupture or sudden death occur relatively often.^2^ Though rare, clinicians should maintain a high-level suspicion for chronic aortic dissection when subtle findings of contrast retention are noted during left heart catheterization. Finally, it is safe to operate in most patients with chronic Type A aortic dissection as they are often haemodynamically stable.^3^

## Supplementary Material

ytaf144_Supplementary_Data

## Data Availability

All data are incorporated into the article.
